# Estimating dementia risk in an African American population using the DCTclock

**DOI:** 10.3389/fnagi.2023.1328333

**Published:** 2024-01-11

**Authors:** Marissa Ciesla, Jeff Pobst, Joyce Gomes-Osman, Melissa Lamar, Lisa L. Barnes, Russell Banks, Ali Jannati, David Libon, Rodney Swenson, Sean Tobyne, David Bates, John Showalter, Alvaro Pascual-Leone

**Affiliations:** ^1^Linus Health, Boston, MA, United States; ^2^Department of Neurology, University of Miami Miller School of Medicine, Miami, FL, United States; ^3^Rush Alzheimer’s Disease Center, Chicago, IL, United States; ^4^Department of Psychiatry and Behavioral Sciences, Rush University Medical Center, Chicago, IL, United States; ^5^Department of Neurological Sciences, Rush University Medical Center, Chicago, IL, United States; ^6^Department of Communicative Sciences and Disorders, College of Arts and Sciences, Michigan State University, East Lansing, MI, United States; ^7^Department of Neurology, Harvard Medical School, Boston, MA, United States; ^8^Department of Geriatrics and Gerontology, New Jersey Institute for Successful Aging, Rowan University School of Osteopathic Medicine, Stratford, NJ, United States; ^9^University of North Dakota School of Medicine and Health Sciences, Fargo, ND, United States; ^10^Hinda and Arthur Marcus Institute for Aging Research and Deanna and Sidney Wolk Center for Memory Health, Hebrew SeniorLife, Boston, MA, United States

**Keywords:** dementia, DCTclock, Alzheimer’s disease, dementia estimation, Rush Alzheimer’s Disease Research Center, Minority Aging Research Study (MARS), African American clinical

## Abstract

The prevalence of Alzheimer’s disease (AD) and related dementias (ADRD) is increasing. African Americans are twice as likely to develop dementia than other ethnic populations. Traditional cognitive screening solutions lack the sensitivity to independently identify individuals at risk for cognitive decline. The DCTclock is a 3-min AI-enabled adaptation of the well-established clock drawing test. The DCTclock can estimate dementia risk for both general cognitive impairment and the presence of AD pathology. Here we performed a retrospective analysis to assess the performance of the DCTclock to estimate future conversion to ADRD in African American participants from the Rush Alzheimer’s Disease Research Center Minority Aging Research Study (MARS) and African American Clinical Core (AACORE). We assessed baseline DCTclock scores in 646 participants (baseline median age = 78.0 ± 6.4, median years of education = 14.0 ± 3.2, 78% female) and found significantly lower baseline DCTclock scores in those who received a dementia diagnosis within 3 years. We also found that 16.4% of participants with a baseline DCTclock score less than 60 were significantly more likely to develop dementia in 5 years vs. those with the highest DCTclock scores (75–100). This research demonstrates the DCTclock’s ability to estimate the 5-year risk of developing dementia in an African American population. Early detection of elevated dementia risk using the DCTclock could provide patients, caregivers, and clinicians opportunities to plan and intervene early to improve cognitive health trajectories. Early detection of dementia risk can also enhance participant selection in clinical trials while reducing screening costs.

## 1 Introduction

The prevalence of Alzheimer’s disease (AD) and related dementias (ADRD) is increasing as the population ages, and its impact will be felt the most by racial and/or ethnic minorities. The older adult African American population is expected to grow from 4.9 million in 2019 to ∼12.1 million by 2060 (US Department of Health and Human Services, 2020). A 2021 survey found that 21.3% of African Americans above the age of 70 are living with ADRD (Alzheimer’s Association, 2021) and estimates suggest that this number will grow to 2.2 million by 2060 (CDC, 2016). African Americans are at higher risk for developing dementia than other ethnic and non-Hispanic White Americans (Mehta and Yeo, 2017), possibly due to a higher prevalence of risk factors including cardiovascular conditions such as diabetes as well as various genetic factors including APOE and ABCA7 that increase dementia risk (Froehlich et al., 2001; Barnes and Bennett, 2014; Alzheimer’s Association, 2021). A recent survey found that 80% of African Americans readily identify barriers to healthcare that negatively impact rates of ADRD diagnosis and access to therapeutic interventions, such as cultural barriers that impede patient-provider relationships and lack of diversity in healthcare (CDC, 2016; Findley et al., 2023). Additionally, 66% of respondents felt it is difficult to get excellent care specific to AD (Alzheimer’s Association, 2021). Despite the fact that African Americans are twice as likely to develop dementia as White Americans (Barnes and Bennett, 2014; Younan et al., 2021), they are 35% less likely to be diagnosed with dementia (Lennon et al., 2022) ADRD identification is further complicated by the current clinical reality where diagnosis typically occurs well after the onset of cognitive symptoms when available treatment options are limited and less effective (Albert et al., 2013; Diogo et al., 2022).

Traditional cognitive screening solutions lack the sensitivity to independently identify individuals at risk for or at the very early stages of cognitive decline and often rely on the administration of additional assessments to confirm the results of cognitive screening tests (Jongstra et al., 2018; Clarke et al., 2022; Thaipisuttikul et al., 2022). Thus, a sensitive method of detecting the early signs of ADRD is urgently needed in order to shift diagnostic and treatment timelines, particularly as it relates to older African Americans. While lower scores on traditional cognitive assessments such as the Montreal Cognitive Assessment (MoCA) and Mini-Mental State Examination (MMSE) are associated with a higher risk of conversion to dementia, clinically meaningful score thresholds and the time course of conversion to dementia are unclear (Arevalo-Rodriguez et al., 2015). Some evidence suggests combining multiple traditional cognitive assessments may improve dementia estimation over time (Masur et al., 1994; Jongstra et al., 2018). However, evidence for the standalone value of traditional pen-and-paper cognitive assessments in estimating dementia risk and conversion over time is limited, as is the practicality of their standardized administration. Digital cognitive assessments can capture emergent cognitive impairment to enhance patient care and also enable more effective, affordable clinical trial selection.

The DCTclock is a digital adaptation of the well-established clock drawing assessment and is an FDA-listed Class II medical device. The DCTclock takes only 3 min to complete and has demonstrated good power to detect cognitive impairment as well as the presence of AD pathology in pre-symptomatic individuals in largely non-diverse samples (Souillard-Mandar et al., 2016, 2021; Rentz et al., 2021). Here, we conducted a retrospective analysis incorporating DCTclock, neuropsychological testing, and clinical diagnostic data from the Rush Alzheimer’s Disease Center Minority Aging Research Study (MARS) (Barnes et al., 2012) and the African American Clinical Core (AACORE). Previous longitudinal analyses of the RUSH ADC and affiliated cohorts have been published demonstrating characteristics associated with conversion to ADRD (Han et al., 2022; Wagner et al., 2022). The present study assesses the ability of the DCTclock to estimate the conversion to ADRD in African Americans within 5 years.

## 2 Methods

### 2.1 Participants

**FIGURE 1:**
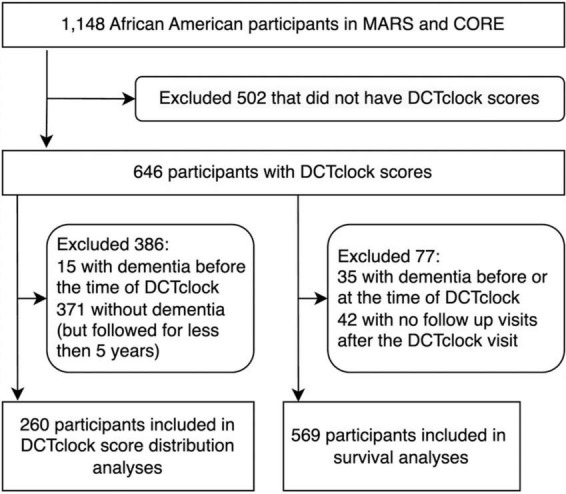
Flow chart of participant inclusion for each set of analyses: DCTclock score distributions and survival analysis.

Data were gathered as part of the Rush Alzheimer’s Disease Center (RADC) Minority Aging Research Study (MARS) (Barnes et al., 2012) and the African American Clinical Core (AACORE). MARS participants were community-dwelling African Americans recruited from churches, subsidized senior housing facilities, retirement communities, African-American clubs, organizations, fraternities, sororities, and social service centers that catered to seniors in the metropolitan Chicago area and outlying suburbs as previously described (Barnes et al., 2012). In the AACORE study, participants were also community-dwelling and were recruited from the metropolitan Chicago area and outlying suburbs, the majority of whom self-identified as Black or African American (Schneider et al., 2009; Melissa et al., 2024) (More information can be found on the RADC website).^1^ Each study was approved by the Institutional Review Board at Rush University Medical Center. Inclusion criteria were adults aged 64 and older who self-identified as Black or African American without known dementia at the time of enrollment. A total of 1,148 African Americans were enrolled in the MARS and AACORE (807 and 341 participants, respectively), out of whom 502 participants were excluded (Figure 1). Of those excluded participants, 126 were male and 376 were female (75% female); the median age at their first visit in the study was 73.39 ± 6.86 and the median years of education was 14.0 ± 3.6. Of those 502, 490 did not take the DCTclock assessment, and 12 individuals took the DCTclock but their results were unanalyzable due to either no detectable clock face or less than 2 non-noise strokes in the drawing (Figure 1). Additionally, participants were excluded from this analysis if they received a dementia diagnosis before their first DCTclock or if they never received a dementia diagnosis but were followed for less than 5 years after their first DCTclock (Figure 1). Thus, in a retrospective analysis, we analyzed longitudinal data from 646 participants (median age at baseline = 78.4 ± 6.5, median years of education = 14.0 ± 3.2, 81% female). All participants provided informed consent prior to participation in the study.

Once a year between 2017 and 2023, participants underwent a full clinical evaluation and completed neuropsychological assessments (Barnes et al., 2012; Han et al., 2022). Clinical assessments were conducted on both cohorts, using harmonized procedures. Briefly, participants were administered a uniform, structured clinical evaluation including a battery of 19 cognitive tests. A neuropsychologist blinded to participant age, gender, and race reviewed the results and other clinical information, and rendered a clinical judgment regarding the presence of dementia. An experienced clinician then reviewed all available data and rendered a final diagnostic classification. The clinical diagnosis of dementia and Alzheimer’s dementia was based on criteria of the joint working group of the National Institute of Neurological and Communicative Disorders and Stroke and the Alzheimer’s Disease and Related Disorders Association (NINCDS/ADRDA) (Bennett et al., 2006; Clifford et al., 2011; McKhann et al., 2011; Barnes et al., 2012). At the same time participants completed their clinical and neuropsychological assessments, they also underwent yearly testing with the DCTclock.

### 2.2 DCTclock

Participants underwent yearly testing with the DCTclock, but the DCTclock performance was not considered during the clinical diagnosis. Using a digital pen (Anoto Inc., Boston, MA, USA) the drawing process during command and copy conditions were analyzed using digital technology and machine learning, and a score was generated (Souillard-Mandar et al., 2016; Piers Ryan et al., 2017). The DCTclock is scored from 0 to 100 with 0 to 59 labeled Cognitively impaired; 60 to 74 labeled Indeterminate or borderline test performance; and 75 to 100 labeled Cognitively unimpaired (Souillard-Mandar et al., 2021). The DCTclock score cutoffs that maximize classification accuracy between cognitively healthy and cognitively impaired were determined in a recent validation study (Souillard-Mandar et al., 2021). The cutoff of 60 for the Cognitively impaired classification was determined by the Youden Index, an optimal cutoff point statistic calculated as sensitivity + specificity−1. The cutoff of 75 for Indeterminate classification was determined via a comparative analysis of possible DCTclock cutoffs to established cutoffs used for MMSE classification to facilitate utility in clinical settings and was selected to match a score cutoff of 28 for the MMSE (Souillard-Mandar et al., 2021).

### 2.3 Statistical analysis

Data were analyzed in Python 3.9.10 using custom code made with NumPy 1.23.4, Pandas 1.4.4, and SciPy 1.11.2. Statistical comparisons were used to determine whether a participant’s DCTclock summary score at baseline was associated with future conversion to dementia. This was done with two different approaches. In the first approach, participants were grouped by the number of years until they would have a dementia diagnosis, including non-converters, and distributions of DCTclock scores were compared by group. This approach had the advantage of being less dependent on the relative distribution of the initial cognitive status of our sample. The second approach included a survival analysis that grouped participants by baseline DCTclock score and had the advantage of being more future-looking and clinically useful. In all analyses, only baseline DCTclock scores were considered.

#### 2.3.1 DCTclock score distributions analysis

For the analysis comparing distributions of DCTclock scores, we grouped participants based on the gap (in years) between completing the DCTclock assessment and receiving a dementia diagnosis. In addition to five groups of participants who were diagnosed with dementia 1, 2, 3, 4, or 5 years after completing their baseline DCTclock, two additional groups were created: those who were diagnosed with dementia at their baseline DCTclock visit, and those who did not convert to dementia for at least 5 years to be used for comparison.

When comparing the distributions of DCTclock scores for these groups, statistical significance was established with a Mann-Whitney *U*-test with Bonferroni’s correction to *p* < 0.05.

#### 2.3.2 Survival analysis

**FIGURE 2:**
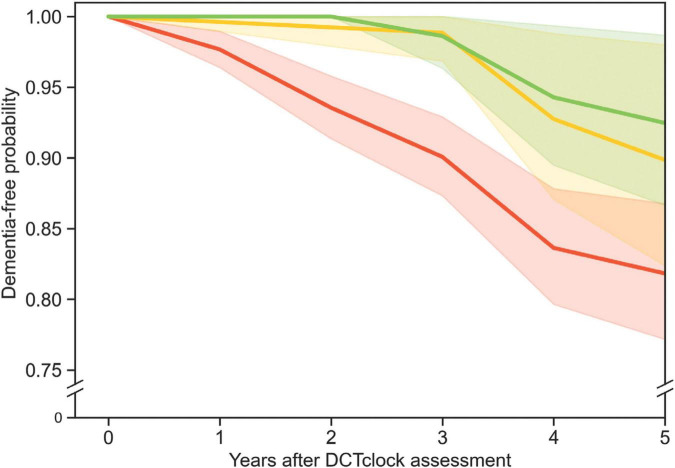
Three survival curves show the probability of participants remaining free from dementia diagnosis over time. The red, yellow, and green curves represent survival curves for three different populations grouped by DCTclock score: those with low (<60), intermediate (≥60, but <75), and high (≥75) scores, respectively. The shaded regions indicate the 90% Greenwood confidence interval for each curve. The green and red curves were significantly different from each other (*p* = 0.014), but the other curve pairs were not.

Survival analysis was used as an estimate analysis as it calculates the probability of a certain outcome (in this case a dementia diagnosis) at various times in the future. Survival analysis was conducted using Python code to implement the Turnbull estimator (Turnbull, 1974) to quantify differences in cognitive health trajectories, i.e., the time from baseline DCTclock performance to conversion to dementia (Figure 2).

Study participants were excluded from the survival analysis if they received a dementia diagnosis at the time of, or at any time prior to, the DCTclock visit. Participants were also excluded if they did not have any follow-up visit after their DCTclock visit (Figure 1). The 569 participants included based on these criteria were then split into three groups according to their DCTclock scores, with 391 participants in the Cognitively impaired group, 87 in the Indeterminate group, and 91 in the Cognitively unimpaired group.

When comparing the survival curves, statistical significance was established using a log-rank chi-square test.

## 3 Results

### 3.1 DCTclock score distribution

**FIGURE 3:**
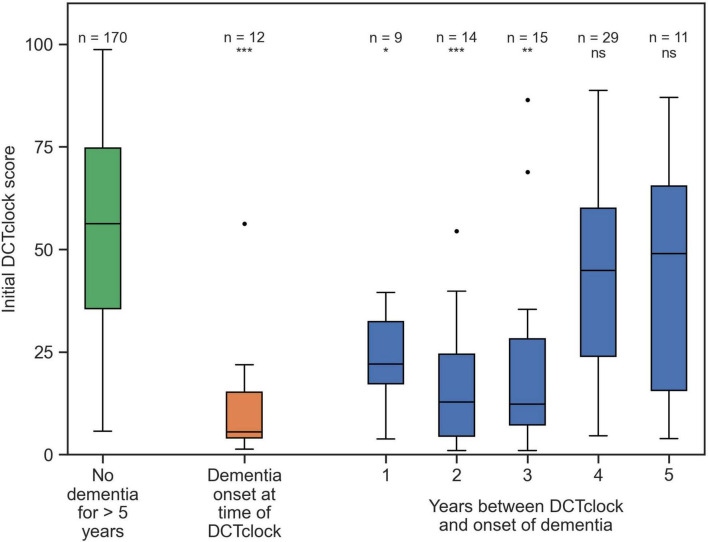
Distributions of DCTclock scores. The green box shows the DCTclock scores for participants who did not get dementia for at least 5 years after completing a DCTclock assessment; the orange box shows the scores for participants who were diagnosed with dementia for the first time in the same visit that they completed the DCTclock, and the blue boxes show the scores of participants who were first diagnosed with dementia some number of years after their baseline visit. Statistical comparisons shown are between the “no dementia” group and each group that would eventually receive a dementia diagnosis. Statistical notation: ns: not significant, *p < 0.01, **p < 0.001, ***p < 0.0001.

Table 1 describes participant demographics at the time of the DCTclock and 5 years after the DCTclock. At the time of their baseline DCTclock, 27 participants had dementia (median age at baseline = 86.5 ± 6.8, median years of education = 14.0 ± 2.9, 70% female) and 619 participants were dementia free (median age at baseline = 78.2 ± 6.3, median years of education = 14.0 ± 3.2, 81% female). A total of 5 years after their baseline DCTclock 78 participants transitioned to dementia (median age at baseline = 87.2 ± 6.6, median years of education = 14.0 ± 3.5, 83% female) while 170 remained dementia free (median age at baseline = 82.0 ± 5.3, median years of education = 15.0 ± 2.0, 88% female). To note, this table does not include those who did not get dementia in years 1, 2, 3, 4 and were lost to follow-up at year 5. The 170 participants who were known not to have dementia for at least 5 years after completing their baseline DCTclock had a mean DCTclock score of 54.2 ± 24.1 (Figure 3). This suggests that many in this group already had some degree of cognitive impairment even though they would not be diagnosed with dementia for more than 5 years. These participants were younger, and had a higher education than those who transitioned to dementia over 5 years. Similarly, participants who received a dementia diagnosis 1 to 5 years after their baseline DCTclock assessment may have also had some degree of cognitive impairment at the time of the baseline DCTclock assessment. The scores of the group who never received a dementia diagnosis for at least 5 years were statistically significantly different from those of the participants that received a dementia diagnosis on the same visit as their DCTclock (mean DCTclock score of 12.2 ± 15.5), 1 year post-baseline (mean DCTclock score of 21.6 ± 12.0), 2 years post-baseline (mean DCTclock score of 17.7.6 ± 16.4), or 3 years post-baseline (mean DCTclock score of 22.2 ± 24.7) with *p* < 0.01 for those groups. The DCTclock scores for participants with onset of dementia 4 years (mean DCTclock score of 43.0 ± 24.6) and 5 years (mean DCTclock score of 42.8. ± 31.2) after the DCTclock test were comparable to the baseline DCTclock scores of the group who did not receive a dementia diagnosis for at least 5 years.

**TABLE 1:** Participant demographics at time of DCTclock and 5 years after DCTclock.

	*n*	Age	Education	% Female
**At time of DCTclock**				
Dementia	27	86.5 ± 6.8	14 ± 2.9	70
Dementia free	619	78.2 ± 6.3	14 ± 3.2	81
**5 years after DCTclock**				
Transitioned to dementia over 5 years	78	87.2 ± 6.6	14 ± 3.5	83
No dementia for >5 years	170	82.0 ± 5.3	15 ± 2.8	88

Data are represented as mean ± SD. To note, the 5 years after DCTclock time-point does not include those who did not get dementia in years 1, 2, 3, 4 and were lost to follow-up at year 5.

### 3.2 Survival analysis

All DCTclock score groups (i.e., Cognitively impaired, Indeterminate, Cognitively unimpaired) contained a portion of participants who converted to dementia within 5 years after taking the baseline DCTclock. The survival analysis included 569 participants (387 from MARS and 182 from AACORE) who were followed for 5 years after their initial DCTclock. The conversion rates over the 5 years of follow-up were 7.7% (7 of 91) for those with a Cognitively unimpaired baseline score, 9.2% (8 of 87) for Indeterminate scorers, and 16.4% (64 of 391) for Cognitively impaired scorers (Figure 2). Those who scored in the cognitively impaired range were significantly more likely to convert to dementia within 5 years than those who scored Cognitively unimpaired (*p* = 0.014). There was no significant difference in the rate of conversion for Indeterminate vs. Cognitively unimpaired scores (*p* = 0.50) or indeterminate vs. Cognitively impaired scores (*p* = 0.33) for this sample population.

## 4 Discussion

The present results demonstrate that baseline DCTclock scores can estimate the risk of dementia conversion in African American individuals, up to 5 years prior to a clinical diagnosis. We show that individuals with a DCTclock score of Cognitively impaired have more than double the chance of developing dementia in 5 years than those with a cognitively unimpaired score. This work provides evidence for the utility of the DCTclock, a 3-min digital cognitive assessment, for early detection of cognitive impairment that can be associated with a future dementia diagnosis in this population years in advance.

Results of previous studies suggest that early detection of ADRD at preclinical stages can impact the patients’ cognitive and functional outcome (Devanand et al., 2008; McDade et al., 2022; Goldstein et al., 2023). Identification of MCI and dementia due to AD requires establishing both cognitive impairment and a positive biomarker status (Albert et al., 2013). Amyloid plaques, consisting of abnormal deposits of amyloid-beta (Aβ) protein, and phosphorylated tau (p-tau) are key biomarkers of AD and play crucial roles in its pathogenesis. The recently approved disease-modifying treatments (DMTs) such as lecanamab (Leqembi^®^) have demonstrated a slowing of cognitive decline with treatment (McDade et al., 2022; van Dyck et al., 2023), and over 130 compounds are currently under investigation at various stages of clinical trials. However, DMTs likely have substantially smaller therapeutic effects at later stages of cognitive decline (McDade et al., 2022). As such, the FDA has approved lecanemab for MCI or mild dementia due to AD (U.S. Food and Drug Administration, 2023). Recent research also demonstrates inherent variability in neuropathology at early stages of cognitive impairment, even among those with subjective cognitive complaints (Piers Ryan et al., 2017; Goldstein et al., 2023). In order to realize the maximum benefit of emerging treatments, detection of cognitive impairment and assessment of risk must occur at early stages of the disease. Our results show that the DCTclock can identify those who may benefit from such treatment several years prior to being diagnosed with dementia.

Besides important implications for patient management, the present results also indicate that the DCTclock can improve participant screening and selection processes for clinical trials. More accurate estimations of who will experience cognitive decline and who will remain healthy over the course of a trial will lead to better participant selection, improving the likelihood of demonstrating efficacy measures. Additionally, replacing more time consuming and costly assessments with the DCTclock can streamline recruitment and reduce trial screening costs. Moreover, detecting emergent cognitive impairment with digital cognitive assessments offers greater opportunity for individuals to participate in clinical trials aimed at finding more effective treatments (Barnes and Bennett, 2014), particularly individuals from racial or ethnic minoritized populations that are often underrepresented in healthcare research. Specifically, the DCTclock can be used as a screening tool to identify African Americans and others who are likely to develop dementia in the coming years who may benefit from a treatment offered in a clinical trial. Establishing the utility of digital cognitive screening like the DCTclock in underrepresented individuals in research is crucial for achieving health equity.

The DCTclock is a highly sensitive, 3-min digital cognitive assessment that detects early signs of cognitive impairment and uses AI-enabled, process-based metrics to analyze an individual’s cognitive performance. These results are consistent with the findings in the Harvard Aging Brain Study (HABS) which found the DCTclock can detect cognitive impairment with high accuracy and correlates with amyloid and tau burden in pre-symptomatic individuals (Rentz et al., 2021). The HABS was conducted with a predominantly White population, so the results of our study extend the evidence for the utility of the DCTclock to African Americans by demonstrating the dementia-forecasting capability of the DCTclock several years prior to a clinical diagnosis. Early identification of individuals with ADRD along with an accurate estimation of their cognitive trajectory over the next several years allows individuals and their families to receive crucial medical, social, and emotional support; enables healthcare professionals to initiate appropriate treatments and interventions in a timely manner that may help slow down the progression of the disease; and provides patients with a better chance at preserving cognitive function and independence for as long as possible (Dubois et al., 2016).

While traditional paper and pencil-based cognitive screening tools can identify current cognitive status, there is little evidence to support their ability to estimate future dementia risk. An updated Cochrane review of 11 studies of 1,569 MCI participants found no substantial evidence that the MMSE could identify those with MCI who would convert to dementia (Arevalo-Rodriguez et al., 2015). Furthermore, researchers also found that the MMSE required additional cognitive assessments for predicting dementia conversion (Jongstra et al., 2018). One study by Julayanont et al. (2015) found that 90.5% of participants with MCI will convert to dementia in 18 months if they have a MoCA total score of less than 20 and a memory index score of less than seven. However, detecting dementia conversion in such a short time prior to dementia onset is insufficient for effective pharmaceutical or lifestyle interventions to be implemented (Kivipelto et al., 2018; van Dyck et al., 2023). Current wait times to be visited by a specialist, can be as much as 9 months. Therefore, a longer prognostic window is required. The DCTclock is a non-invasive, brief assessment that produces immediate, objective, and reliable results to providers and can be used to understand dementia conversion risk up to 5 years earlier than traditional cognitive testing. Furthermore, the DCTclock is an easy-to-use clinical evaluation tool that easily integrates into primary care and specialist workflows including electronic health records, which eases the burden on time-constrained healthcare professionals. Thus, leveraging digital assessment platforms, like the DCTclock, built with sensitive, process-based, metrics that can detect signs of cognitive impairment and estimate dementia risk, allowing for more immediate interventions aimed at reducing dementia risk while improving function.

This study is limited, in part, by the characteristics of the sample population. First, the sample was primarily female (78%), but it should be noted that rates of female representation in dementia clinical trials favor females as do rates of dementia diagnosis (Hale et al., 2020; Pinho-Gomes et al., 2022). Also of note, in comparison to previously published work, the Rush sample used in this study had a lower baseline DCTclock performance than expected, particularly in those who were never diagnosed with cognitive impairment during the course of the study. Future studies should examine additional available cognitive assessments to evaluate subtle cognitive impairment or interactions with demographic characteristics such as education or social determinants of health including experiences of discrimination. Additionally, this study is limited by the number of conversions (78) to dementia over the period studied, as presented in the survival curves. This number is not large enough to enable the training validated, predictive regression models that account for multiple covariates without quickly encountering overfitting effects. Future studies will aim to acquire additional data that will allow for multiple covariates to estimate dementia risk. Lastly, comprehensive life and health data were not collected as part of this study which would have provided a holistic summary of an individual’s dementia risk. Future work aims to identify these dementia risk factors and use them to further understand longitudinal dementia risk using the DCTclock.

This research demonstrates the ability of the DCTclock to estimate the 5-year risk of developing dementia in an African American population. Early detection of future dementia risk using the DCTclock can open doors for early initiation of therapies such as anti-amyloid-drugs and dementia clinical trials. It can also streamline participant selection in clinical trials while reducing screening costs. Additionally, early detection of dementia risk with the DCTclock can provide patients, caregivers, and clinicians the opportunity to plan ahead and intervene early to improve the patients’ cognitive trajectories.

## Data availability statement

The datasets presented in this study can be found in online repositories. The names of the repository/repositories and accession number(s) can be found below: https://www.radc.rush.edu/home.htm.

## Ethics statement

The studies involving humans were approved by the Institutional Review Board at Rush University Medical Center. The studies were conducted in accordance with the local legislation and institutional requirements. Written informed consent was obtained from all participants prior to study procedures.

## Author contributions

MC: Conceptualization, Formal analysis, Writing – original draft, Writing – review and editing. JP: Formal analysis, Writing – original draft, Writing – review and editing, Data curation. JG-O: Formal analysis, Writing – original draft, Writing – review and editing. ML: Data curation, Funding acquisition, Writing – review and editing. LB: Data curation, Funding acquisition, Writing – review and editing. RB: Formal analysis, Writing – original draft, Writing – review and editing. AJ: Formal analysis, Writing – review and editing. DL: Writing – review and editing. RS: Writing – review and editing. ST: Formal analysis, Writing – review and editing, Data curation. DB: Conceptualization, Writing – review and editing. JS: Writing – review and editing. AP-L: Formal analysis, Writing – review and editing, Conceptualization.

## Footnotes

1
https://www.radc.rush.edu/docs/parentStudyDesigns.htm
